# Metabolite markers for three synthetic tryptamines *N*‐ethyl‐*N*‐propyltryptamine, 4‐hydroxy‐*N*‐ethyl‐*N*‐propyltryptamine, and 5‐methoxy‐*N*‐ethyl‐*N*‐propyltryptamine

**DOI:** 10.1002/dta.3668

**Published:** 2024-03-09

**Authors:** Marianne Skov‐Skov Bergh, Inger Lise Bogen, Katharina Elisabeth Grafinger, Marilyn A. Huestis, Åse Marit Leere Øiestad

**Affiliations:** ^1^ Section for Drug Abuse Research, Department of Forensic Sciences, Division of Laboratory Medicine Oslo University Hospital Oslo Norway; ^2^ Section for Pharmacology and Pharmaceutical Biosciences, Department of Pharmacy, The Faculty of Mathematics and Natural Sciences University of Oslo Oslo Norway; ^3^ Institute of Chemistry and Bioanalytics University of Applied Sciences and Arts Northwestern Switzerland Muttenz Switzerland; ^4^ Institute of Emerging Health Professions Thomas Jefferson University Philadelphia Pennsylvania USA; ^5^ Section for Forensic Toxicological Analytics, Department of Forensic Sciences, Division of Laboratory Medicine Oslo University Hospital Oslo Norway

**Keywords:** 4‐hydroxy‐*N*‐ethyl‐*N*‐propyltryptamine (4‐OH‐EPT), 5‐methoxy‐*N*‐ethyl‐*N*‐propyltryptamine (5‐MeO‐EPT), metabolite, microsomes, *N*‐ethyl‐*N*‐propyltryptamine (EPT), new psychoactive substances (NPS), synthetic tryptamines, ultra‐high performance liquid chromatography–quadrupole time‐of‐flight mass spectrometry (UHPLC‐QTOF‐MS)

## Abstract

*N*‐Ethyl‐*N*‐propyltryptamine (EPT), 4‐hydroxy‐*N*‐ethyl‐*N*‐propyltryptamine (4‐OH‐EPT), and 5‐methoxy‐*N*‐ethyl‐*N*‐propyltryptamine (5‐MeO‐EPT) are new psychoactive substances classified as tryptamines, sold online. Many tryptamines metabolize rapidly, and identifying the appropriate metabolites to reveal intake is essential. While the metabolism of 4‐OH‐EPT and 5‐MeO‐EPT are not previously described, EPT is known to form metabolites by indole ring hydroxylation among others. Based on general knowledge of metabolic patterns, 5‐MeO‐EPT is also expected to form ring hydroxylated EPT (5‐OH‐EPT). In the present study, the aim was to characterize the major metabolites of EPT, 4‐OH‐EPT, and 5‐MeO‐EPT, to provide markers for substance identification in forensic casework. The tryptamines were incubated with pooled human liver microsomes at 37°C for up to 4 h. The generated metabolites were separated and detected by ultra‐high performance liquid chromatography–quadrupole time‐of‐flight mass spectrometry analysis. The major in vitro EPT metabolites were formed by hydroxylation, *N*‐dealkylation, and carbonylation. In comparison, 4‐OH‐EPT metabolism was dominated by double bond formation, *N*‐dealkylation, hydroxylation, and carbonylation in vitro and hydroxylation or carbonylation combined with double bond loss, carbonylation, *N*‐dealkylation, and hydroxylation in vivo. 5‐MeO‐EPT was metabolized by *O*‐demethylation, hydroxylation, and *N*‐dealkylation in vitro. The usefulness of the characterized metabolites in forensic casework was demonstrated by identification of unique metabolites for 4‐OH‐EPT in a human postmortem blood sample with suspected EPT or 4‐OH‐EPT intoxication.

## Graphical abstract

We characterized the major metabolites of the synthetic tryptamines EPT, 4‐OH‐EPT, and 5‐MeO‐EPT after incubation with pooled human liver microsomes. The usefulness of the discovered metabolites in forensic casework was demonstrated by identification of unique metabolites for 4‐OH‐EPT in a human postmortem blood sample with suspected EPT or 4‐OH‐EPT intoxication.

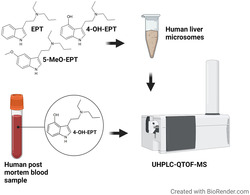

## 1 INTRODUCTION

**FIGURE 1:**
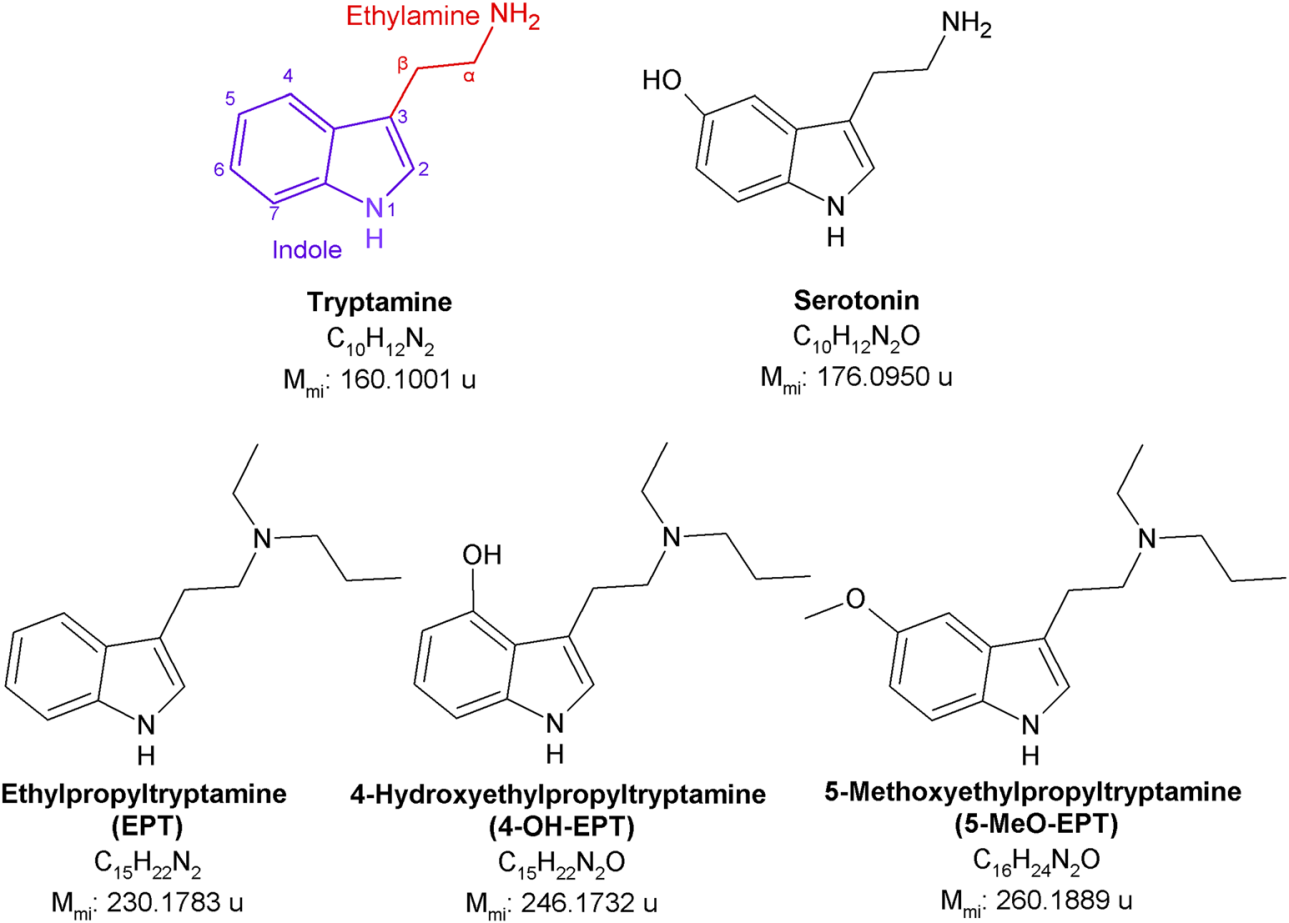
Structural formulas, chemical formulas, and monoisotopic mass (*M*_mi_) of tryptamine, serotonin, EPT, 4‐OH‐EPT, and 5‐MeO‐EPT. The tryptamine core structure is identical to that of the neurotransmitter serotonin, consisting of an indole ring and an amino group, connected with an ethyl spacer.

The presence of synthetic tryptamines on the illicit drug market is currently increasing, with 57 compounds monitored by the European Monitoring Centre for Drug and Drug Addiction (EMCDDA) as of October 2022.^1^ Clandestine laboratories generate new synthetic tryptamines by introducing minor changes to the tryptamine core structure (Figure 1), mainly in positions four and five of the indole ring as this increases potency.^2^, ^3^ Synthetic tryptamines can be administered by various routes (orally, injected, smoked, or insufflated)^3^, ^4^, ^5^ and mediate their hallucinogenic effects by activation of serotonin receptors (5‐HT_2A_).^6^, ^7^, ^8^, ^9^ Several tryptamines require minor doses in the low mg range to induce psychotropic effects^3^, ^5^, ^10^ and have a delayed onset, which increases the risk of repeated administration, potentially leading to intoxications or fatalities.^3^, ^4^, ^10^, ^11^, ^12^, ^13^ The fact that synthetic tryptamine users often are unaware of the true identity and potency of the purchased compound further increases the risk of accidental overdose.^4^ *N*,*N*‐Dialkylated tryptamine intake is also dangerous because altered states of consciousness may lead to dangerous or irrational behavior.^2^, ^5^, ^10^ These inherit dangers make synthetic tryptamine analysis important in forensic toxicology laboratories. However, bioanalysis can be challenging due to intake of low doses, rapid metabolism, and instability in blood and solution.^5^, ^14^, ^15^, ^16^, ^17^, ^18^ Indole ring hydroxylated tryptamines can also degrade under the influence of air and light.^16^, ^19^ In forensic casework, the ingested compound may not be detectable, making identification of metabolites essential for confirming synthetic tryptamine intake.^20^, ^21^, ^22^

*N*‐Ethyl‐*N*‐propyltryptamine (EPT), 4‐hydroxy‐*N*‐ethyl‐*N*‐propyltryptamine (4‐OH‐EPT), and 5‐methoxy‐*N*‐ethyl‐*N*‐propyltryptamine (5‐MeO‐EPT) are three structurally similar synthetic tryptamines (Figure 1) currently available on the illicit drug market. 4‐OH‐EPT and EPT were seized in Japan between 2017 and 2019^23^ and in Europe in 2018 and 2019,^24^ respectively. Meanwhile, 5‐MeO‐EPT was first detected in Japan in 2008.^25^ The pharmacology of these compounds was subject to limited research and publication. However, a recent study on structure–activity relationships of tryptamines^26^ reported that 4‐OH‐EPT is a highly efficacious 5‐HT_2A_ and 5 HT_2B_ agonist with a potency in the low nanomolar range, similar to psilocin^26^ and LSD.^27^ 4‐OH‐EPT was also reported to bind to a number of other receptors and targets (5HT_1_, 5HT_5–7_, H_1_, D_2_, Alpha_2A_, NR2B, SERT, and Sigma 1 and 2).^28^ To the best of the authors' knowledge, the metabolic pathways for 4‐OH‐EPT and 5‐MeO‐EPT are not reported. In a previous study examining the metabolism of EPT in human liver S9 fraction and EPT‐exposed rats,^22^ EPT metabolites formed by *N*‐dealkylation, *N*‐oxide formation, alkyl hydroxylation, indole ring hydroxylation, and indole ring hydroxylation followed by glucuronidation or sulfation were reported, with no detectable parent compound. Because indole ring hydroxylation is a major pathway for EPT, 4‐OH‐EPT is a potential metabolite. 5‐MeO‐EPT is also expected to form ring hydroxylated EPT because 5‐methoxylated tryptamines are known to be metabolized mainly through *O*‐demethylation, generating the 5‐OH metabolite.^12^, ^21^, ^29^, ^30^, ^31^, ^32^, ^33^ 4‐OH‐EPT can thus be a metabolite of EPT, and EPT and 5‐MeO‐EPT may form a shared metabolite, making them difficult to discern, especially in cases where the ingested substance is undetectable.

The aim of this study was to discover the major metabolites of EPT, 4‐OH‐EPT, and 5‐MeO‐EPT in order to provide markers for substance identification in forensic casework. The metabolites were formed using pooled human liver microsomes (pHLM) and detected by ultra‐high performance liquid chromatography–quadrupole time‐of‐flight mass spectrometry (UHPLC‐QTOF‐MS) analysis. The usefulness of the characterized metabolites in forensic casework was demonstrated by identification of unique metabolites for 4‐OH‐EPT in a human postmortem blood sample with suspected EPT or 4‐OH‐EPT intoxication.

## 2 MATERIALS AND METHODS

### 2.1 Chemicals, reagents, and materials

EPT (98%) and 5‐MeO‐EPT hydrochloride (99.4%) were purchased from Cayman Chemicals (Ann Arbor, MI, USA). 4‐OH‐EPT (98%) was purchased from Chiron AS (Trondheim, Norway). Psilocin‐D_10_ and amphetamine‐D_11_ were purchased from Cerilliant (Round Rock, TX, USA) and formic acid (98%), ammonium formate, and ethyl acetate from VWR International AS (Leuven, Belgium). Chromasolv methanol (MeOH) of LC–MS grade was purchased from Honeywell Riedel‐de Haën (Seelze, Germany). pHLM (XTreme 200 Pool) were from XenoTech (Kansas City, KS, USA) and nicotinamide adenine dinucleotide phosphate (NADPH) regeneration solutions A (NADP^+^ and glucose‐6‐phosphate) and B (glucose‐6‐phosphate dehydrogenase) were purchased from Corning Incorporated (Corning, NY, USA). Disodium tetraborate decahydrate and heptane were from Chemi‐Teknik AS (Oslo, Norway). Type 1 water (18.2 MΩ) was purified with a Synthesis A 10 milli‐Q system from Millipore (Billerica, MA, USA). Blank human ante mortem whole blood for calibrators and quality control (QC) samples was obtained from the blood bank at Oslo University Hospital (Oslo, Norway). Heparin (final conc. 17 IU/mL; Leo Pharma, Lysaker, Norway) and sodium fluoride (final conc. 4 mg/mL, Merck KGaA, Darmstadt, Germany) were added to the blood as anticoagulant and preservative, respectively.

### 2.2 In vitro drug metabolism studies in pHLM

pHLM from 200 donors of balanced gender (480 pmol total cytochrome P450/mg protein) were stored in a 250 mM sucrose solution at −80°C. pHLM (final concentration 2 mg protein/mL) and NADPH regeneration solution (final concentration 1.3 mM NADP+, 3.3 mM glucose‐6‐phosphate, 0.4 U/mL glucose‐6‐phosphate dehydrogenase, 3.3 mM MgCl_2_) were added to amber‐colored Eppendorf tubes and pre‐incubated at 37°C for 10 min in a shaking water bath protected from light. The reaction was initiated by addition of EPT, 4‐OH‐EPT, or 5‐MeO‐EPT (final concentration 10 μM) and stopped after 0, 60 or 240 min by addition of ice‐cold formic acid (final concentration 0.1 M) followed by immediate vortexing. Tubes were centrifuged at 4°C and 14,500 × *g* for 10 min and supernatants transferred to auto sampler vials placed on ice. Control samples for drug substance degradation (in the absence of pHLM), and negative pHLM control samples (not added drug substance), were performed in parallel and stopped after 0 and 240 min. All pHLM incubations were carried out in duplicate in two independent experiments, and the samples analyzed immediately by UHPLC‐QTOF‐MS.

### 2.3 Sample preparation of a human postmortem blood sample

During routine work at the Department of Forensic Sciences, Oslo University Hospital, a human postmortem blood sample was positive for 4‐OH‐EPT by UHPLC‐QTOF‐MS screening. The blood sample was subsequently quantified by UHPLC‐MS/MS. The recorded QTOF‐MS data from the blood sample were further examined for metabolites. The data on the postmortem sample are presented with approval from the Norwegian Higher Prosecution Authority (reference number 2021/1321). No case report information is included to ensure the anonymity of the sample donor.

A human postmortem blood sample was collected from the femoral vein and stored in a 25 mL Steriline tube (Cole‐Parmer, Staffordshire, United Kingdom) containing 200 mg potassium fluoride as a preservative. For routine screening by UHPLC‐QTOF‐MS, the postmortem blood sample was prepared by liquid–liquid extraction. The blood sample (500 μL) was transferred to a sample tube and amphetamine‐D_11_ internal standard (0.25 μM prepared in water, 50 μL) was added. Borate buffer (pH 11, 250 μL) was added before vortexing. Ethyl acetate/heptane (1200 μL, 4 + 1, v/v) was added, followed by vortexing and 10 min light mechanical turning of the sample. The tube was centrifuged for 15 min at 4°C at 5200 × *g*. The supernatant was evaporated to dryness at 40°C under a stream of nitrogen gas. The sample was reconstituted in ammonium formate (pH 3.1)/MeOH (100 μL, 4 + 1, v/v), vortexed, and transferred to an autosampler vial before untargeted UHPLC‐QTOF‐MS analysis.

For quantification, 100 μL postmortem blood was added to a sample tube, and 50 μL ammonium formate (pH 3.1)/MeOH (9 + 1, v/v) and 50 μL 20 μM psilocin‐D_10_ internal standard were added before vortexing. The sample was prepared as described above for the UHPLC‐QTOF‐MS screening with minor adjustments. Borate buffer (100 μL) was added to the tube before vortexing and addition of ethyl acetate/heptane (1000 μL), followed by mechanical turning and centrifugation. The supernatant was evaporated and the sample reconstituted in ammonium formate (pH 3.1)/MeOH (100 μL, 9 + 1 v/v) before targeted UHPLC‐MS/MS analysis. Five calibrators (0.1–2 μM) and two QC samples (0.15 and 1.5 μM) of 4‐OH‐EPT were prepared in human ante mortem whole blood and extracted and analyzed simultaneously.

### 2.4 UHPLC‐QTOF‐MS and UHPLC‐MS/MS analysis

pHLM samples and the postmortem blood sample were analyzed by a UHPLC‐QTOF‐MS method described previously,^34^ as well as in the Data S1. The QTOF data were analyzed by Masshunter Qualitative Analysis (version B.08.00, Agilent Technologies) combined with a Personal Compound Database Library (PCDL) containing phases I and II biotransformations predicted by Metabolite Tool 2.0 (Broeckers Solutions, Berlin, Germany) as well as known biotransformations of tryptamines. The following search criteria were used: a minimum peak height of 5000 counts after 60 min of incubation with pHLM, a maximum mass error of 10 ppm, a maximum of 10 matches per formula, and a chromatogram extraction window of maximum 100 ppm. The following criteria were used for metabolite identification: a symmetrical peak shape, no co‐eluting peaks, a retention time fitting the proposed metabolite (compared with the retention time of the parent drug), increasing peak height if identical peaks were present in the control samples for drug substance degradation, no presence of identical peaks in the negative pHLM control samples, a maximum mass error of 5 ppm for the protonated molecule, and a MS/MS fragmentation pattern in accordance with the proposed metabolite structure.

4‐OH‐EPT was quantified in the extracted postmortem blood sample by targeted UHPLC‐MS/MS analysis with an Acquity UPLC™ (Waters, Milford, MA, USA) coupled to a Xevo‐TQS triple quadrupole MS with an electrospray ionization interface (Waters). Chromatographic separation was performed using a 9 min gradient described previously,^35^ as well as in Data S2, on an Acquity BEH C_18_ column (2.1 × 50 mm, 1.7 μm, Waters, Wexford, Ireland) at 65°C and a mobile phase consisting of 5 mM ammonium formate (pH 10.2) (solvent A) and MeOH (solvent B) at a flow rate of 0.5 mL/min. The injection volume was 0.5 μL. MS/MS analysis was performed using positive ionization and multiple‐reaction monitoring (MRM) mode. Data acquisition and processing were performed using Masslynx™ 4.1 software (Waters). The MRM transitions and MS/MS parameters were *m/z* 247.4 > 160.0 and *m/z* 247.4 > 100.0 for 4‐OH‐EPT and *m/z* 215.1 > 164.0 for the internal standard psilocin‐D_10_. Cone voltage and collision energy was 15 V and 20 eV, respectively, for both compounds. Retention times were 3.09 and 1.69 min for 4‐OH‐EPT and psilocin‐D_10_, respectively. 4‐OH‐EPT was quantified in the sample using a weighted calibration curve (1/*x*) excluding the origin, constructed by plotting five calibrator concentrations against analyte/IS peak height ratio.

## 3 RESULTS AND DISCUSSION

### 3.1 Fragmentation patterns of the parent compounds

**FIGURE 2:**
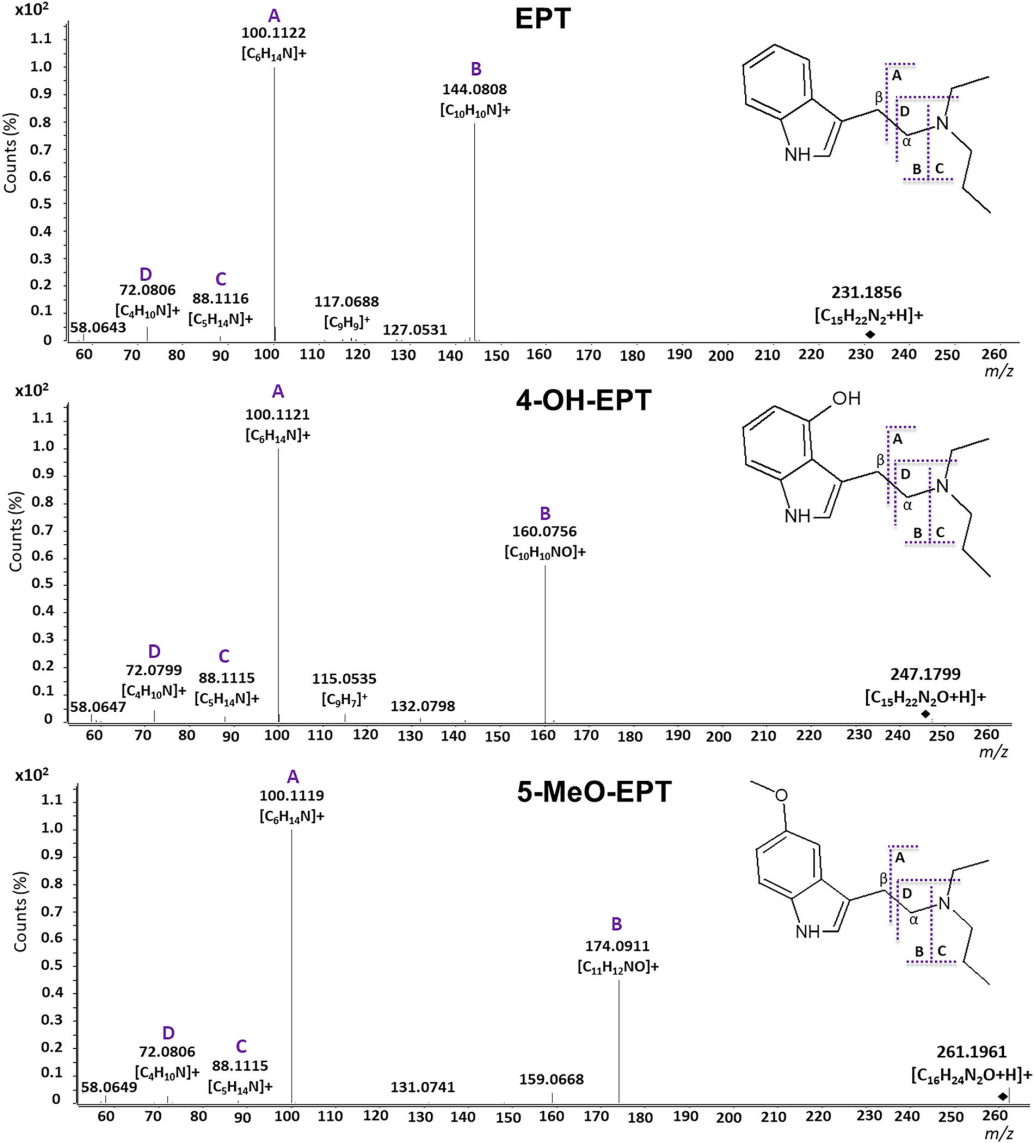
MS/MS spectra of EPT, 4‐OH‐EPT, and 5‐MeO‐EPT, as well as proposed fragmentation pattern for the compounds. Characteristic fragments used for the identification of metabolites are labeled A, B, C, and D, respectively. Fragments A, C, and D were identical for all three tryptamines; however, fragment B was dependent on the parent tryptamine. The protonated molecular ions are marked by black tiles.

The aim of this study was to characterize the major metabolites of EPT, 4‐OH‐EPT, and 5‐MeO‐EPT in order to provide markers for detection of the ingested substance in forensic casework. The fragmentation patterns of the parent compounds, determined by QTOF‐MS analysis of single component drug solutions, were used to elucidate structures of the formed metabolites. The MS/MS spectra of EPT, 4‐OH‐EPT, and 5‐MeO‐EPT, and proposed fragmentation patterns are displayed in Figure 2. All masses given in the following sections are the calculated exact masses.

Figure 2 shows that fragmentation of the protonated EPT (*m/z* 231.1856), 4‐OH‐EPT (*m/z* 247.1805), and 5‐MeO‐EPT (*m/z* 261.1961) generated a base peak corresponding to fragment A (*m/z* 100.1121), which originates from an α‐cleavage of the ethyl linker connecting the indole structure and amine moiety. The second most abundant peak for all three compounds was fragment B (*m/z* 144.0808, 160.0757, or 174.0913), resulting from an amine‐cleavage between the amine moiety and ethyl linker, which gave a unique fragment for each tryptamine. When the charge remained on the opposite side of the fragmentation site, this cleavage also generated fragment C (*m/z* 88.1121). Fragment D (*m/z* 72.0808), corresponding to secondary fragmentation of fragment A by a neutral loss of ethene, was also observed in the spectra of EPT, 4‐OH‐EPT, and 5‐MeO‐EPT. Additionally, a fragment (*m/z* 117.0699, C_9_H_9_^+^) representing the indole ring and β‐carbon after loss of HCN was found in the spectrum of EPT. A similar fragment (*m/z* 115.0542, C_9_H_7_^+^) corresponding to the indole ring with β‐carbon after loss of HCN and further elimination of water was found in the spectrum of 4‐OH‐EPT.

### 3.2 Identification of metabolites after incubation with pHLM

**FIGURE 3:**
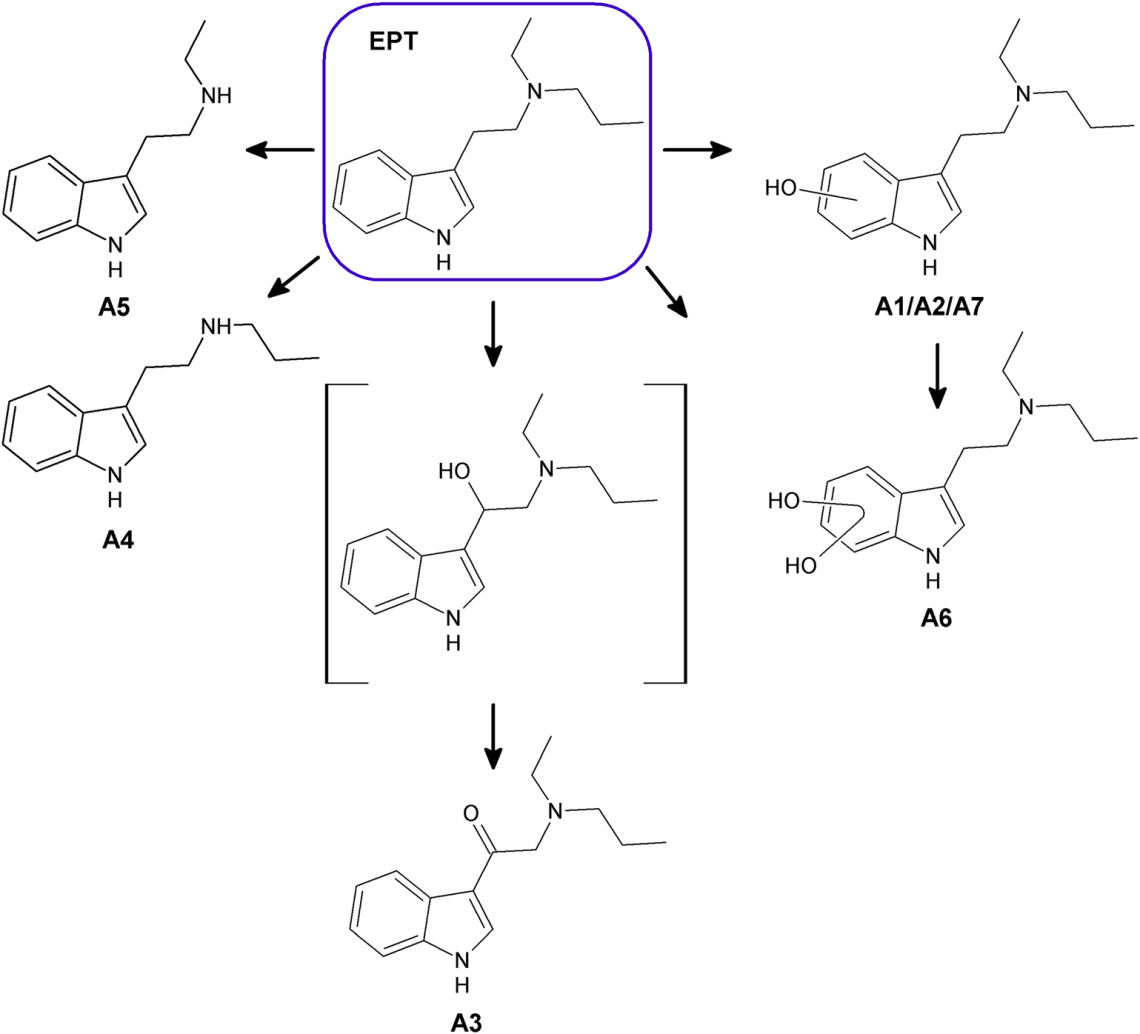
Main metabolites of EPT identified after incubation with pHLM (A1–A7). Markush bonds indicate possible locations for hydroxyl groups. In brackets: intermediate of the formed metabolite which was not found in the samples.

**FIGURE 4:**
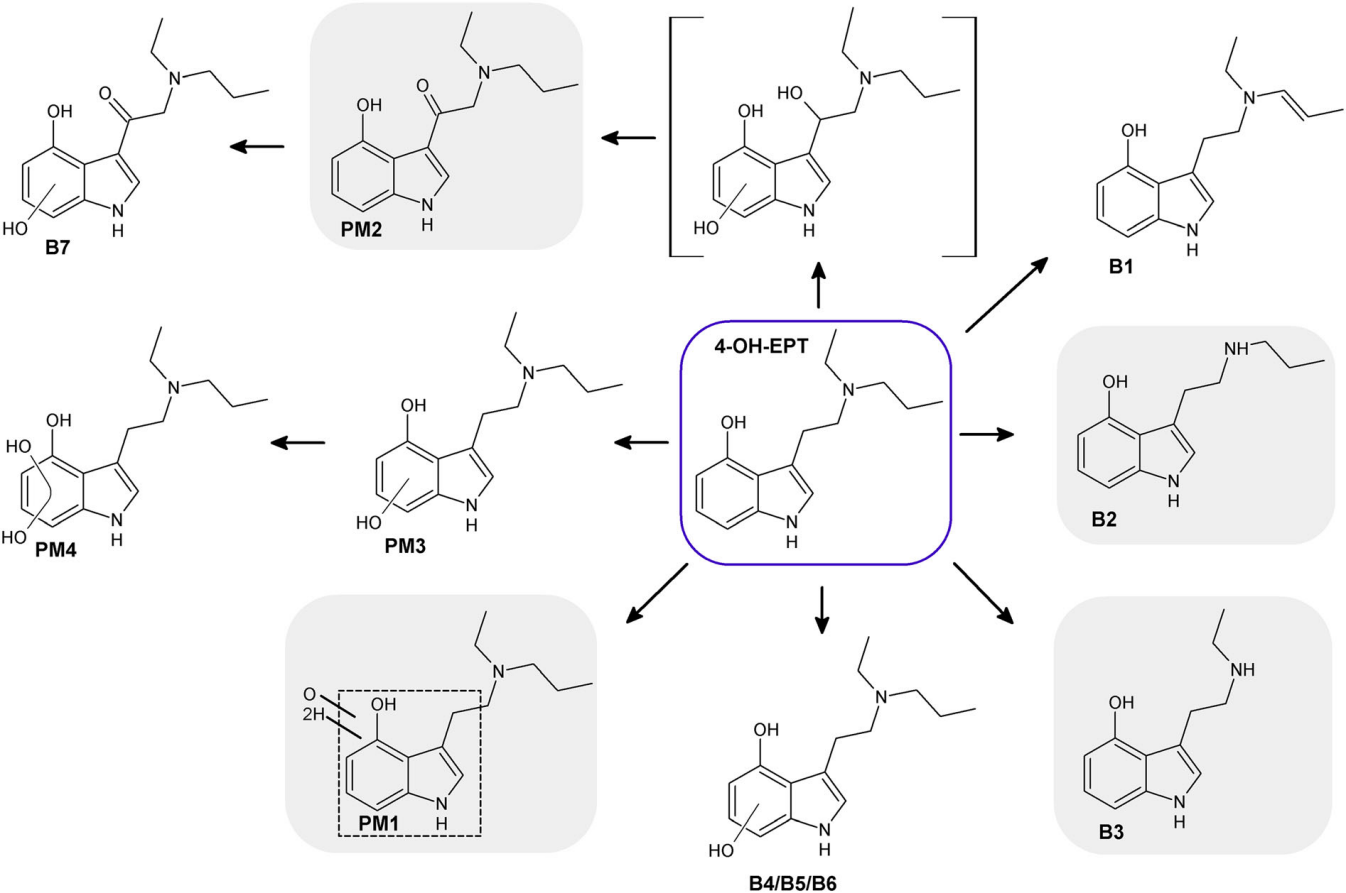
Main metabolites of 4‐OH‐EPT identified after incubation with pHLM (B1–B7) and in a postmortem blood sample suggesting 4‐OH‐EPT intoxication (PM1–PM4). Markush bonds indicate possible locations for oxygen, hydrogen atoms, or a hydroxyl or carbonyl group. In grey squares: formed both in vitro and in vivo. In brackets: intermediate of the formed metabolite which was not found in the samples. In dotted square: the exact biotransformation could not be determined.

The six to seven most abundant metabolites of EPT, 4‐OH‐EPT, and 5‐MeO‐EPT formed after 60 min, but also detectable after 240 min incubation with pHLM, were characterized to potentially serve as metabolite markers in forensic case work. Possible metabolite structures were elucidated by comparing the fragmentation patterns of the parent compounds (Figure 2) with those of the metabolites. The identified metabolites of EPT, 4‐OH‐EPT, and 5‐MeO‐EPT are listed in Tables 1 and 2, and the proposed structures are shown in Figure 3, 4, 5. MS/MS spectra and extracted ion chromatograms of the metabolites are depicted in the Supporting nformation.

**TABLE 1:** Proposed main metabolites of EPT (A1–A7), 4‐OH‐EPT (B1–B7), and 5‐MeO‐EPT (C1–C6) in pHLM and of 4‐OH‐EPT in a postmortem blood sample (PM1–PM4, B2, and B3) with biotransformation, retention time (*T*_R_), molecular formula, accurate mass of protonated molecule, mass error, and experimental average mass of MS/MS product ions.

Drug	ID	Biotransformation	*T*_R_ (min)^a^	Molecular formula	[M + H]^+^ (*m/z*)^b^	Mass error (ppm)^c^	MS/MS product ions^d^
EPT	P^e^		5.92	C_15_H_22_N_2_	231.1856	1.5	**144.0808**, 117.0691, ***100.1121***, 88.1119, 72.0804, 58.0647
	A1	Hydroxylation	3.89	C_15_H_22_N_2_O	247.1805	−1.3	**160.0752**, ***100.1123***, 72.0802, 58.0646
	A2	Hydroxylation	4.64	C_15_H_22_N_2_O	247.1805	−1.2	**160.0756**, 132.0801, ***100.1119***, 88.1121, 72.0798, 58.0647
	A3	Carbonylation	4.51	C_15_H_20_N_2_O	245.1648	−1.8	216.1255, 202.1099, 187.0866, 173.0700, **160.0756**, ***100.1120***,
	A4	*N*‐Deethylation	5.23	C_13_H_18_N_2_	203.1543	3.9	***144.0808***, **72.0802**, 132.0803
	A5	*N*‐Depropylation	4.15	C_12_H_16_N_2_	189.1386	3.3	***144.0807***, **58.0647**, 132.0803
	A6	Dihydroxylation	3.38	C_15_H_22_N_2_O_2_	263.1754	−2.2	***176.0703***, **148.0390**, 100.1115, 88.1119
	A7	Hydroxylation	4.17	C_15_H_22_N_2_O	247.1805	−1.6	***160.0752***, 132.0801, 132.0440, **100.1118**, 88.1117, 72.0799
4‐OH‐EPT	P^e^		4.75	C_15_H_22_N_2_O	247.1805	−2.9	**160.0756**, 132.0800, 115.0541, ***100.1121***, 88.1117, 72.0803, 58.0648
	B1^f^	Double bond formation	4.39	C_15_H_20_N_2_O	245.1648	−1.2	***216.1257***, 215.1169, ***202.1100***, 187.0861, 173.0701, 160.0756, 146.0598
	B2	*N*‐Deethylation	3.84	C_13_H_18_N_2_O	219.1492	−1.4 (0.2)	***160.0755***,148.0755, 115.0529, **72.0805**, 58.0646
	B3	*N*‐Depropylation	2.85	C_12_H_16_N_2_O	205.1335	−2.1 (−0.1)	***160.0755***, 148.0751, 115.0539, **58.0648**
	B4	Hydroxylation	4.24	C_15_H_22_N_2_O_2_	263.1754	−2.7	***176.0705***, 148.0755, **100.1117**, 88.1115, 72.0803
	B5	Hydroxylation	3.80	C_15_H_22_N_2_O_2_	263.1754	−3.0	***176.0704***, 148.0744, **148.0388**, 100.1114, 88.1116
	B6	Hydroxylation	3.43	C_15_H_22_N_2_O_2_	263.1754	−2.6	***176.0704***, 148.0752, 148.0386, **100.1117**, 88.1114
	B7	Carbonylation and hydroxylation	4.61	C_15_H_20_N_2_O_3_	277.1547	−2.0	**190.0499**, 162.0547, 149.0233, ***100.1117***, 88.1115, 72.0798
	PM1	Hydroxylation or carbonylation combined with double bond loss	4.03	C_15_H_24_N_2_O_2_	265.1911	−0.4	**178.0862**, 160.0760, ***100.1124***, 72.0800
	PM2	Carbonylation	5.29	C_15_H_20_N_2_O_2_	261.1598	−0.8	***174.0543***, **100.1120**, 88.1108, 58.0643
	PM3	Hydroxylation	3.93	C_15_H_22_N_2_O_2_	263.1754	−0.2	**178.0859**, 176.0699, 160.0753, 148.0382, ***100.112***, 88.1125
	PM4	Dihydroxylation	4.27	C_15_H_22_N_2_O_3_	279.1703	−0.1	**164.0707**, 146.0603, ***100.1120***, 72.0814, 58.0641
5‐MeO‐EPT	P^e^		5.90	C_16_H_24_N_2_O	261.1961	−3.2	**174.0909**, ***100.1118***, 88.1112, 72.0802, 58.0647
	C1	*O*‐Demethylation	3.70	C_15_H_22_N_2_O	247.1805	−1.6	**160.0756**, ***100.1121***, 88.1120, 72.0804, 58.0645
	C2	Hydroxylation	4.37	C_16_H_24_N_2_O_2_	277.1911	1.4	190.0864, 115.0538, ***100.1121***, **72.0802**, 58.0664
	C3	*N*‐Deethylation	5.30	C_14_H_20_N_2_O	233.1648	−1.3	***174.0910***, 162.0907, **72.0803**
	C4	*N*‐Depropylation	4.39	C_13_H_18_N_2_O	219.1492	1.8	***174.0911***, 162.0911, **58.0648**
	C5	Hydroxylation	4.66	C_16_H_24_N_2_O_2_	277.1911	1.0	**190.0859**, 162.0536, ***100.1118***, 72.0803
	C6	Hydroxylation	4.94	C_16_H_24_N_2_O_2_	277.1911	2.4	***190.0863***, 162.0538, **100.1121**, 88.1118

*Note*: The metabolites are sorted after peak height measured in pHLM (60 min) or in the postmortem blood sample.

Abbreviation: P, parent compound.

^a^ Mean *T*_R_ measured in pHLM and drug degradation control samples (without pHLM) or *T*_R_ measured in the postmortem blood sample.

^b^ Exact *m/z* of the protonated molecule.

^c^ The highest mass error obtained for pHLM samples (60 and 240 min) or the postmortem blood sample. Mass error in parentheses is for metabolites detected in the blood sample.

^d^ Average of the MS/MS product ions detected at all time points in pHLM samples, or MS/MS product ions detected in the postmortem blood sample, with base peaks (bold italics) and second most abundant peaks (bold) highlighted.

^e^ Signal intensity for parent compound saturated for several drug degradation control and pHLM samples.

^f^ Equally abundant base peaks.

**TABLE 2:** Proposed main metabolites of EPT (A1–A7), 4‐OH‐EPT (B1–B7), and 5‐MeO‐EPT (C1–C6) in pHLM and of 4‐OH‐EPT in a postmortem blood sample (PM1–PM4, B2, and B3) with retention time (*T*_R_) error and peak height in drug degradation control samples (without pHLM), pHLM, and in a postmortem blood sample.

Drug	ID	Biotransformation	*T*_R_ ^a^ error sample	Peak height in drug degradation control samples (cps)^b^		Peak height in pHLM (cps)^b^			Peak height in postmortem blood sample (cps)
				0 min	240 min	0 min	60 min	240 min	
EPT	P			3.5 × 10^6^	3.4 × 10^6^	3.5 × 10^6^	3.2 × 10^6^	1.8 × 10^6^	
	A1	Hydroxylation		ND	ND	ND	3.3 × 10^6^	3.3 × 10^6^	
	A2	Hydroxylation		ND	ND	1.1 × 10^4^	3.3 × 10^6^	2.7 × 10^6^	
	A3	Carbonylation		ND	ND	ND	2.2 × 10^6^	2.6 × 10^6^	
	A4	*N*‐Deethylation		1.0 × 10^4^	1.1 × 10^4^	1.1 × 10^4^	1.7 × 10^6^	3.3 × 10^4^	
	A5	*N*‐Depropylation		6.3 × 10^3^	6.5 × 10^3^	6.6 × 10^3^	8.3 × 10^5^	7.3 × 10^3^	
	A6	Dihydroxylation		ND	ND	ND	6.8 × 10^5^	1.1 × 10^6^	
	A7	Hydroxylation		2.1 × 10^4^	2.5 × 10^4^	6.0 × 10^4^	4.9 × 10^5^	4.4 × 10^5^	
4‐OH‐EPT	P		0.14	3.4 × 10^6^	3.6 × 10^6^	3.4 × 10^6^	3.5 × 10^6^	3.4 × 10^6^	4.0 × 10^6^
	B1	Double bond formation		4.3 × 10^3^	3.3 × 10^3^	6.8 × 10^3^	1.6 × 10^6^	1.9 × 10^6^	ND
	B2	*N*‐Deethylation	0.15	1.7 × 10^4^	1.5 × 10^4^	1.7 × 10^4^	1.5 × 10^6^	1.5 × 10^6^	2.7 × 10^5^
	B3	*N*‐Depropylation	0.12	1.3 × 10^4^	1.1 × 10^4^	1.3 × 10^4^	1.3 × 10^6^	1.3 × 10^6^	4.7 × 10^4^
	B4^c^	Hydroxylation		ND	6.5 × 10^4^	ND	6.4 × 10^5^	9.7 × 10^4^	ND
	B5	Hydroxylation		2.3 × 10^4^	6.3 × 10^4^	2.1 × 10^5^	4.7 × 10^5^	9.3 × 10^5^	ND
	B6	Hydroxylation		1.1 × 10^4^	3.3 × 10^4^	1.3 × 10^5^	3.8 × 10^5^	6.2 × 10^5^	ND
	B7	Carbonylation and hydroxylation		ND	ND	ND	2.6 × 10^5^	2.2 × 10^5^	ND
	PM1	Hydroxylation or carbonylation combined with double bond loss	0.12	ND	ND	ND	5.2 × 10^3^	7.7 × 10^3^	4.6 × 10^5^
	PM2	Carbonylation	0.15	ND	ND	8.8 × 10^4^	3.3 × 10^4^	2.3 × 10^4^	5.6 × 10^4^
	PM3	Hydroxylation	‐	ND	ND	ND	ND	ND	4.7 × 10^4^
	PM4	Dihydroxylation	‐	ND	ND	ND	ND	ND	4.6 × 10^4^
5‐MeO‐EPT	P			3.6 × 10^6^	3.5 × 10^6^	3.6 × 10^6^	3.5 × 10^6^	3.4 × 10^6^	
	C1	*O*‐Demethylation		ND	ND	ND	3.2 × 10^6^	3.4 × 10^6^	
	C2	Hydroxylation		ND	ND	ND	1.8 × 10^6^	1.7 × 10^6^	
	C3	*N*‐Deethylation		2.1 × 10^4^	2.2 × 10^4^	2.2 × 10^4^	1.6 × 10^6^	1.4 × 10^5^	
	C4	*N*‐Depropylation		1.1 × 10^4^	1.1 × 10^4^	1.1 × 10^4^	4.6 × 10^5^	2.7 × 10^4^	
	C5	Hydroxylation		3.2 × 10^4^	3.3 × 10^3^	8.8 × 10^4^	3.3 × 10^5^	4.7 × 10^5^	
	C6	Hydroxylation		3.1 × 10^4^	3.7 × 10^4^	9.4 × 10^4^	1.8 × 10^5^	7.5 × 10^4^	

^a^ Difference in *T*_R_ between the postmortem blood sample and pHLM samples (measurements were performed at different time points).

^b^ Average peak height for each time point (*n* = 4).

^c^ Also formed by degradation during incubation.

Because of the reported instability of several tryptamines,^5^, ^14^, ^15^, ^16^, ^17^, ^18^ efforts were made to protect experimental samples from air and light. Several compounds were found, which did not meet the criteria described previously, for metabolites during the PCDL search (data not shown). These compounds were most likely impurities stemming from the synthesis process (purity was 98% to 99.4%) or degradation products formed during solution storage, and are not further discussed.

### 3.3 EPT metabolites

The seven most abundant EPT metabolites formed after incubation with pHLM, A1–A7, are listed in Tables 1 and 2 sorted after peak height measured in the pHLM incubated for 60 min, and the proposed structures are shown in Figure 3. The relative abundance of the metabolites was A1 = A2 > A3 > A4 > A5 > A6 > A7 after 60 min and A1 > A2 > A3 > A6 > A7 > A4 > A5 after 240 min incubation with pHLM.

Three metabolites, namely, metabolite A1, A2, and A7 (*m/z* 247.1805), were formed by EPT hydroxylation because the mass corresponded to EPT plus oxygen (15.9949 u). Fragment A was the base peak in the MS/MS spectra of A1, A2, and A7, and the presence of a fragment corresponding to fragment B plus oxygen (*m/z* 160.0757), indicated that the hydroxylation takes place on the indole ring or the β‐carbon of the ethyl linker. EPT metabolites hydroxylated at the β‐position are previously reported to generate water loss fragments with *m/z* 229.1699.^22^ The lack of such a water loss fragment in the spectra of all three metabolites indicates that the hydroxylation is localized at the indole ring. This is further supported by the presence of a fragment corresponding to the hydroxylated indole ring (*m/z* 132.0444) in the spectra of A2 and A7.

Metabolite A3 (*m/z* 245.1648) was formed by carbonylation because the *m/z* corresponds to the mass of EPT plus oxygen minus two hydrogens (2.0157 u). The carbonyl is suggested to be located at the β‐carbon because carbonylation at the indole ring would generate a metabolite with a different *m/z* (247.1805). Fragment A was the most abundant peak in the A3 spectrum, followed by a peak corresponding to protonated fragment B plus oxygen minus two hydrogens (*m/z* 160.0757, C_10_H_10_NO^+^). The observation of fragments generated by loss of ethene (*m/z* 216.1257) and propene (*m/z* 202.1101) from the substituents of the amino group further supports that the change occurred at the β‐carbon and not on the amine moiety.

Metabolite A4 (*m/z* 203.1543) and A5 (*m/z* 189.1386) were formed by *N*‐deethylation and *N*‐depropylation of EPT, because the masses correspond to EPT minus C_2_H_4_ (28.0313 u) and C_3_H_6_ (42.0470 u), respectively. This is further supported by the fragment B base peak and the absence of fragments A and C. Fragment D was the second most abundant fragment in the spectra of A4, whereas *m/z* 58.0651, corresponding to a similar fragmentation of *N*‐depropylated EPT, was the second most abundant fragment of A5. A fragment corresponding to a β‐cleavage with the charge retained on the indole moiety (*m/z* 132.0808, C_9_H_10_N^+^) was also observed in the spectra of A4 and A5.

Metabolite A6 (*m/z* 263.1754) was formed by dihydroxylation at the indole ring because the mass corresponds to EPT plus two oxygens (31.9898 u) and a fragment corresponding to the indole ring moiety plus two oxygens (*m/z* 148.0393, C_8_H_6_NO_2_^+^) was observed in the spectrum. The most abundant peak was fragment B plus two oxygens (*m/z* 176.0706), and fragments A and C were also observed.

Metabolites A4, A5, and A7 were also present in the control samples for drug degradation (not added pHLM) with peak heights similar to those observed in pHLM incubation samples at *t* = 0 (Table 2). It was therefore concluded that these compounds are not solely metabolites but also degradation products or synthesis impurities. Such compounds were reported previously for EPT.^22^ The peak heights for A4, A5, and A7 in the control samples were low (<2% of EPT peak) and did not increase in the drug degradation control samples during the 240 min incubation, indicating that the compounds were not generated by thermal degradation during the experiment.

### 3.4 4‐OH‐EPT metabolites

The seven most abundant metabolites of 4‐OH‐EPT generated after the incubation with pHLM, B1–B7, are listed in Tables 1 and 2 sorted after peak height measured in the pHLM incubated for 60 min, and suggested metabolite structures are shown in Figure 4. The relative metabolite abundance was B1 > B2 > B3 > B4 > B5 > B6 > B7 after 60 min and B1 > B2 > B3 > B5 > B6 > B7 > B4 after 240 min incubation with pHLM.

Metabolite B1 (*m/z* 245.1648) displayed a mass corresponding to 4‐OH‐EPT minus two hydrogens, indicating double bond formation. The presence of fragment B and a fragment (*m/z* 146.0600, C_9_H_8_NO^+^) corresponding to an α‐cleavage with the charge remaining on the β‐carbon side, as well as trace amounts of fragment A, indicated an alteration in the amine moiety. The most abundant peaks (*m/z* 216.1257 and *m/z* 202.1101) were generated by ethene loss from the amine moiety and ethene loss followed by additional fragmentation of the propyl substituent, respectively. The high abundance of *m/z* 202.1101 implies that metabolism of the propyl substituent formed an enamine. Fragments corresponding to additional breakdown of the amine moiety (*m/z* 215.1179, 187.0866, and 173.0709) were also detected.

Metabolites B2 (*m/z* 219.1494) and B3 (*m/z* 205.1339) were formed by *N*‐deethylation and *N*‐depropylation of 4‐OH‐EPT, because the masses correspond to 4‐OH‐EPT minus C_2_H_4_ and C_3_H_6_, respectively, and the fragmentations patterns resemble those of the *N*‐dealkylated metabolites of EPT (A4 and A5) shifted by one oxygen.

Metabolites B4–B6 (*m/z* 263.1754) were generated by hydroxylation at the indole ring because the masses corresponded to 4‐OH‐EPT plus oxygen and the fragmentation patterns were similar to the indole ring dihydroxylated EPT metabolite A6. The observation of a fragment (*m/z* 148.0393, C_8_H_6_O_2_N^+^) corresponding to a dihydroxylated indole moiety in the spectra of metabolites B5 and B6 supported this.

Metabolite B7 (*m/z* 277.1547) is suggested to be formed by hydroxylation and carbonylation with an *m/z* corresponding to the mass of 4‐OH‐EPT plus two oxygens minus two hydrogens. The most abundant peaks were fragment A and fragment B plus two oxygens minus two hydrogens (*m/z* 190.0499). Fragments C and D were also present in the spectra. Altogether this indicated that the biotransformation took place on the β‐carbon or the indole ring. The presence of a fragment (*m/z* 162.0550, C_9_H_8_NO_2_) corresponding to the indole ring with β‐carbon plus one oxygen, and the lack of water loss fragments as seen for EPT hydroxylated at the β‐carbon^22^ indicated hydroxylation of the indole ring. This suggests that B7 corresponds to carbonylation at the β‐carbon of the ethyl linker and hydroxylation at the indole ring.

Similar to EPT, several 4‐OH‐EPT metabolites (B1–B3, B5, and B6) were also present in the control samples and pHLM samples prior to incubation (Table 2). These compounds can also be degradation products or synthesis impurities. Minor amounts of metabolite B4 was also generated by degradation during incubation of 4‐OH‐EPT.

### 3.5 5‐MeO‐EPT metabolites

**FIGURE 5:**
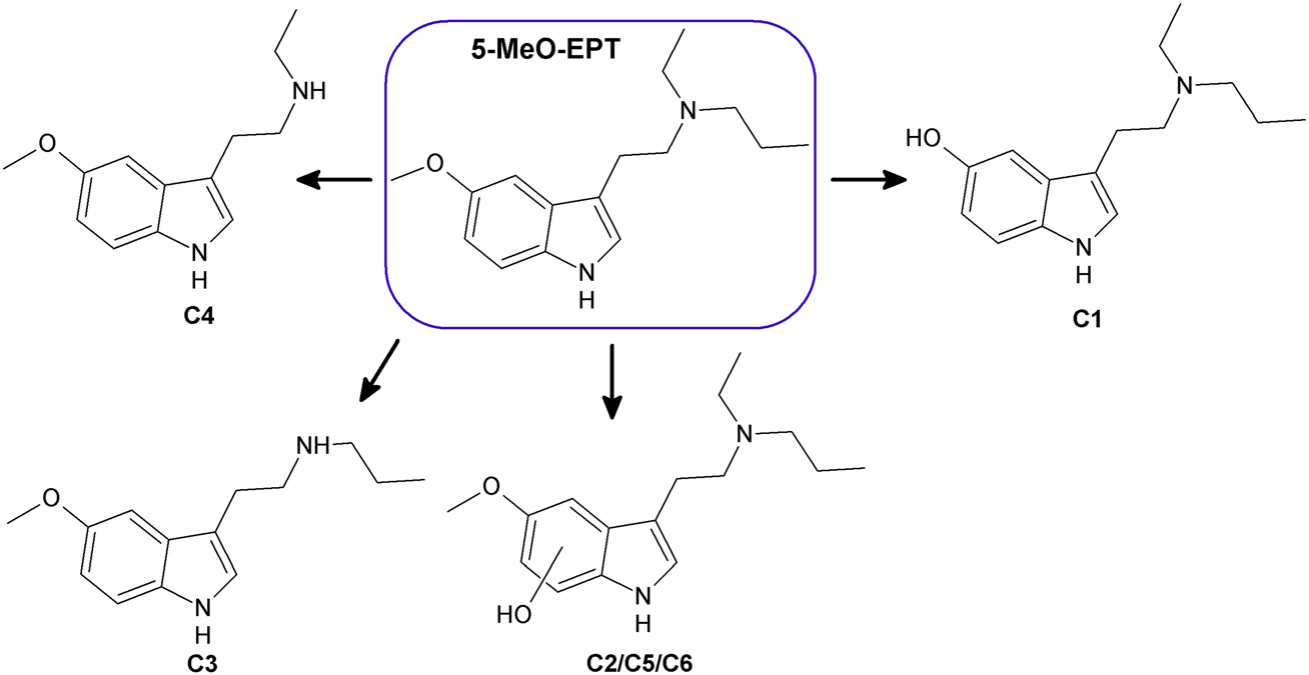
Main metabolites of 5‐MeO‐EPT identified after incubation with pHLM (C1–C6). Markush bonds indicate possible locations for a hydroxyl group.

The six most abundant metabolites of 5‐MeO‐EPT formed after incubation with pHLM, C1–C6, are listed in Tables 1 and 2 sorted after peak height measured in the pHLM incubated for 60 min, and the proposed structures are shown in Figure 5. The relative abundance of the metabolites was C1 > C2 > C3 > C4 > C5 > C6 after 60 min and C1 > C2 > C5 > C3 > C6 > C4 after 240 min incubation with pHLM.

Metabolite C1 (*m/z* 247.1805) was formed by demethylation of the methoxy group because the mass corresponds to 5‐MeO‐EPT minus one carbon (12.0000 u) and two hydrogens. The base peak corresponding to fragment B minus one carbon and two hydrogens, as well as the presence of fragment A, C, and D, indicate that demethylation occurs at the 5‐methoxy substituent of the indole ring (*O*‐demethylation).

Metabolite C2, C5, and C6 (*m/z* 277.1911) were generated by hydroxylation of the indole ring because the masses correspond to 5‐MeO‐EPT plus oxygen, and the fragmentation patterns were similar to the indole hydroxylated metabolites of EPT (A1, A2, and A7) and 4‐OH‐EPT (B4–B6) shifted by one methoxy or methyl group. The presence of a fragment ion (*m/z* 162.0550, C_9_H_8_NO_2_) corresponding to the methoxylated indole ring plus oxygen in the spectra of C5 supported this. A fragment (*m/z* 162.0925) corresponding to the protonated indole ring moiety was also observed in the spectra of C6. A highly abundant fragment with *m/z* 149.0233 (C_8_H_5_O_3_^+^) of unknown origin sporadically appeared in the spectra of C2, C5, and C6. Compounds with *m/z* 149.0233, or a base peak at 149.0233, eluted in close proximity to the metabolites in both the drug degradation control samples (without pHLM) and drug‐negative control samples (containing pHLM only). Consequently, it is inferred that this fragment originates from the instrument background signal.

Metabolites C3 (*m/z* 233.1648) and C4 (*m/z* 219.1492) were produced by *N*‐deethylation and *N*‐depropylation as the masses correspond to 5‐MeO‐EPT minus C_2_H_4_ and C_3_H_6_, respectively, and the fragmentation patterns resemble those of the *N*‐dealkylated metabolites of EPT (A4 and A5) and 4‐OH‐EPT (B2 and B3) shifted by one methoxy or methyl group. The fragment ion (*m/z* 162.0913) corresponding to the protonated indole ring moiety was observed in the spectra as well.

The metabolites C3–C6 were found in minor amount in the drug degradation control samples of 5‐MeO‐EPT (Table 2), suggesting that these metabolites are degradation products or synthesis impurities as well.

### 3.6 Comparison of the metabolism of the three different tryptamine analogues

The major metabolic pathways in pHLM for the three tryptamine analogues EPT, 4‐OH‐EPT, and 5‐MeO‐EPT differed slightly; however, the three compounds also displayed shared biotransformations. All three analogues were metabolized by *N*‐dealkylation at the amine moiety (A4, A5, B2, B3, C3, and C4) and monohydroxylation of the indole ring (A1, A2, A7, B4–B6, C2, C5, and C6). These biotransformations were previously reported for several other tryptamine analogues.^12^, ^20^, ^21^, ^29^, ^32^, ^36^, ^37^, ^38^, ^39^ Metabolism by double bond formation was also seen for 4‐OH‐EPT (B1) in the study. To the best of the authors' knowledge, the metabolic pathway of double bond formation was not reported previously for tryptamines. Metabolism involving carbonylation, which is proposed for A3 and B7, was previously observed for a number of synthetic tryptamines.^20^, ^21^, ^32^, ^37^, ^38^ In this study, 5‐MeO‐EPT metabolism was dominated by *O*‐demethylation of the methoxy group. This biotransformation was previously reported for several other tryptamines with a 5‐MeO group.^12^, ^21^, ^29^, ^30^, ^31^, ^32^, ^33^

All EPT metabolites, with the exception of A3, were in accordance with the metabolites found by Manier et al. in urine from EPT‐exposed rats and in human liver S9 fraction incubated with EPT.^22^ Multiple indole ring hydroxylated EPT metabolites were detected by Manier et al. but could not be definitively identified due to lack of reference standards. Because the 4‐OH‐EPT reference standard was available in the present study, the EPT metabolite A2 could be identified as 4‐OH‐EPT based on *m/z*, fragmentation pattern, and retention time (retention time difference 0.09 min). The dihydroxylated EPT metabolite A6 was also most likely identical to the hydroxylated 4‐OH‐EPT metabolite B6, because of the matching *m/z* ratio, the retention time (difference 0.05 min), and the fragmentation pattern for the two compounds. The major metabolite of 5‐MeO‐EPT, C1, was generated by *O*‐demethylation, producing 5‐OH‐EPT, which potentially could confirm and identify one of the hydroxylated EPT metabolites in the study. However, no metabolite displayed the exact same retention time and fragmentation pattern as C1. To unequivocally establish the correct structure for all the different metabolites, reference standards are required.

### 3.7 Identifying tryptamine analogue metabolites in a postmortem blood sample

In a peripheral postmortem blood sample, untargeted UHPLC‐QTOF‐MS forensic toxicology screening at Oslo University Hospitals' laboratory indicated 4‐OH‐EPT consumption. To confirm and quantify 4‐OH‐EPT, the postmortem blood sample was prepared by liquid–liquid extraction and analyzed by targeted UHPLC‐MS/MS. 4‐OH‐EPT with a concentration of 94 ng/mL was identified in the sample based on the presence of the molecular ion at the correct retention time (<0.16% deviation compared with calibrators and QC samples), the presence of two MRM transitions, and the correct ratio of these ions to one another (5.7% deviation compared with calibrators and QC samples). The difference (residual) between the concentration predicted by the linear calibration curve (*R* ^2^ = 0.999) and the nominal concentration at the calibration points and in the QC samples was no more than ±15%. The method was not validated further. No other tryptamines were detected in the sample. However, because 4‐OH‐EPT is also a metabolite of EPT, a study was undertaken to examine whether the metabolite profile present in the sample could reveal if the deceased ingested EPT or 4‐OH‐EPT. A database search for metabolites in the untargeted UHPLC‐QTOF‐MS screening data was performed. The six most abundant metabolites identified are listed in Tables 1 and 2, and the proposed structures are shown in Figure 4. Extracted ion chromatograms and MS/MS spectra of the metabolites are depicted in the Supporting Information. The relative abundance of 4‐OH‐EPT and metabolites in the postmortem blood sample was 4‐OH‐EPT > PM1 > B2 > PM2 > PM3 = B3 > PM4.

Based on the metabolism study in pHLM, A3 is a suitable marker for distinguishing EPT from 4‐OH‐EPT because this metabolite was not detected following 4‐OH‐EPT intake. Additionally, the *N*‐dealkylated metabolites A4 and A5 are good supportive markers because it is unlikely that the *N*‐dealkylated 4‐OH‐EPT undergoes dehydroxylation rendering the compound less hydrophilic. Because neither A3, A4, nor A5 was detected in the postmortem blood sample, it was concluded that the ingested compound was most likely 4‐OH‐EPT. The 4‐OH‐EPT metabolites detected, and their relative abundance, varied between the blood sample and the samples incubated with pHLM. Some metabolites were also found exclusively in the blood sample. Differences between in vitro and in vivo findings are expected, because in vitro experiments using pHLM are a simplified model of human metabolism. Additionally, there is possible postmortem redistribution in autopsy samples. Of the seven most abundant metabolites detected after incubation in pHLM, only the 4‐OH‐EPT metabolites B2 and B3 were also present in the blood sample (Table 2) and were identified based on matching fragmentation patterns and similar retention times (retention time difference ≤0.2 min). The relative abundance was B2 > B3 both in the blood sample and in the incubated pHLM samples. Several other metabolites were found in the blood sample (PM1–PM4). These metabolites, except PM3 and PM4, were also found after pHLM incubation but were not among the major metabolites identified.

Metabolite PM1 (*m/z* 265.1916) had a mass corresponding to 4‐OH‐EPT plus two hydrogens and one oxygen that indicates a hydroxylation or a carbonylation combined with a loss of a double bond. The presence of fragment B plus two hydrogens and one oxygen, as well as fragment A, placed the biotransformation at the indole ring. A fragment (*m/z* 160.0757) corresponding to water loss from the transformed indole ring followed by re‐aromatization was also observed in the PM1 spectrum. However, the structure of PM1 could not be elucidated conclusively because the fragmentation pattern gave limited data (Figure 4). Carlier et al. published a study examining the metabolism of the structurally similar tryptamine 4‐hydroxy‐*N*,*N*‐methylpropyltryptamine in human liver hepatocytes and reported a metabolite with the same mass shift from the parent compound and similar fragmentation pattern as PM1.^40^ Similar to the presented study, no structural formula was proposed for the metabolite due to inconclusive fragmentation data. However, Carlier et al. proposed that the metabolite could have been formed by ring opening at the α‐carbon of the hydroxyindole moiety generating a carboxylic acid. To elucidate the exact structure for PM1, the metabolite would have to be extracted from the postmortem sample and analyzed using nuclear magnetic resonance spectroscopy.

Metabolite PM2 (*m/z* 261.1598) was formed by carbonylation displaying a mass corresponding to 4‐OH‐EPT plus oxygen minus two hydrogens. The two largest peaks in the PM2 spectrum corresponded to fragment B plus oxygen minus two hydrogens (*m/z* 174.0550) and fragment A. The presence of fragment A indicates that the carbonylation takes place on the β‐carbon. An indole ring hydroxylated metabolite, PM3, with a retention time similar to B5 (retention time difference 0.13 min), was also detected in the sample. However, B5 and PM3 were not the same compound because their fragmentation patterns did not match.

A dihydroxylated metabolite, PM4 (*m/z* 279.1703), displaying a mass corresponding to 4‐OH‐EPT plus two oxygens was also found in the sample. The base peak being fragment A and the presence of fragment D and *m/z* 58.0651 (C_3_H_8_N) indicate that the amine moiety was unchanged. The presence of a fragment (*m/z* 164.0706, C_9_H_10_NO_2_^+^) corresponding to an α‐cleavage with the charge retained on the hydroxyindole moiety plus oxygen, as well as a fragment corresponding to water loss from the previously mentioned fragment (*m/z* 146.0600, C_9_H_8_NO^+^), indicated that one of the hydroxylations occurred at the indole ring. Because fragment A is the base peak and there are no fragments corresponding to water loss at the ethyl linker (parent ion minus water, C_15_H_20_N_2_O_2_^+^), the second hydroxylation most likely also took place at the indole ring. The highly abundant fragment with an *m/z* of 149.0233 (C_8_H_5_O_3_^+^) that appeared sporadically in the spectra of C2, C5, and C6 was also seen in the spectrum of PM4. This fragment is inferred to originate from the background signal of the instrument.

### 3.8 Metabolite markers for detecting drug intake

With the marked increase in the number of synthetic tryptamines, metabolite identification may be essential to distinguish the ingested tryptamine in forensic casework. Generally, a good marker of drug intake should be abundant and specific for the parent compound by not losing substantial parts of the original structure. Furthermore, the chosen markers for EPT, 4‐OH‐EPT, and 5‐MeO‐EPT should not be shared by the three compounds.

As previously discussed, A3 is a suitable marker for EPT because it is highly abundant and retains the parent drug carbon skeleton. However, it is important to evaluate fragmentation patterns carefully to ensure correct identification because 4‐OH‐EPT forms a metabolite (B1) with the same *m/z*. A4 and A5 are also good candidates for distinguishing intake of EPT from 4‐OH‐EPT and 5‐MeO‐EPT because they are unlikely to be formed from the latter. However, because A4 and A5 do not retain the original structure of EPT, they can only be used as supportive markers. A1 can also be used as a marker because it contains the whole structure of the parent drug and is highly abundant, but it should be combined with a supportive marker to avoid confusion with 4‐OH‐EPT or *O*‐demethylated 5‐MeO‐EPT.

The most abundant metabolite in the pHLM samples, B1, is a marker for the identification of 4‐OH‐EPT intake but should be used with care because the EPT marker A3 has the same *m/z*. B1 was not found in the postmortem blood sample and due to inter‐individual enzyme variations, more in vivo samples are required to examine the prevalence of the metabolite in authentic samples. Metabolites PM1 and B7 could be good markers because they were not found in the pHLM samples incubated with EPT and 5‐MeO‐EPT. However, PM1 might be a better choice because it was detected in a real case sample and is more abundant.

In order to confirm 5‐MeO‐EPT ingestion, the hydroxylated metabolites C2, C5, and C6 should be used as markers as they include the original structure of 5‐MeO‐EPT. However, their presence must be interpreted with caution because hydroxylated metabolites with the same *m/z* were found in the 4‐OH‐EPT and EPT samples. Metabolite C3 can be used as a supportive marker together with the hydroxylated markers, as it was not identified in pHLM samples incubated with EPT or 4‐OH‐EPT. C4 may also be a supportive marker but should be used with care because metabolites with the same *m/z*, but deviating retention times and fragmentation patterns, are formed from both EPT (not among the seven most abundant) and 4‐OH‐EPT (B2).

A weakness of the proposed metabolite markers in this study is the discrepancy between observations made in the in vitro and in vivo models. Such differences were also reported in a metabolism study on 4‐OH‐*N*‐methyl‐*N*‐ethyltryptamine (4‐OH‐MET),^36^ which implies that in vitro results must be interpreted cautiously. The use of pHLM is a complementary model to in vivo data because they alone cannot be used for metabolite marker predictions. Additionally, because superoxide dismutase was not used in the study, it cannot be ruled out that some of the identified metabolites were formed by superoxide radicals. However, most of the metabolites found for EPT were also identified by Manier et al.,^22^ who performed a metabolism study on EPT using superoxide dismutase. Another weakness of this study is that only one authentic case sample was available. More samples are needed to make a definite recommendation on the most suitable 4‐OH‐EPT marker, due to variations in cytochrome P450 in a population, time elapsed from intake to sampling, and postmortem redistribution. The usefulness of the markers detected for EPT and 5‐MeO‐EPT after pHLM incubation could not be evaluated because no information was found on their biotransformation in authentic case samples. Another weakness of the study is that the tentative identification of the metabolites is based on high resolution mass spectrometric data only. Complete determination of the structures of these metabolites is only possible through synthesis and characterization of all the metabolites, including the potential structural isomers, for example, by chromatographic separation, identification using nuclear magnetic resonance spectroscopy and comparison of these with the metabolites detected in vitro. Additionally, co‐elution of structural isomers with similar spectra such as hydroxylated metabolites cannot be excluded because only one column and gradient was utilized. In certain cases, structural isomers might be indistinguishable by UHPLC, and alternative techniques such as capillary electrophoresis are needed in order to separate them.

## 4 CONCLUSION

*N*,*N*‐Dialkylated tryptamines represent an analytical and interpretative challenge in forensic toxicology due to their low concentrations in biological samples, instability, and extensive metabolism. The major in vitro metabolites of the synthetic tryptamine analogues EPT, 4‐OH‐EPT, and 5‐MeO‐EPT formed after incubation with pHLM were identified. The usefulness of the characterized metabolites was demonstrated by identification of unique in vivo metabolites for 4‐OH‐EPT in a human postmortem blood sample with suspected EPT or 4‐OH‐EPT intoxication. Finally, metabolite markers for EPT, 4‐OH‐EPT, and 5‐MeO‐EPT suitable to confirm intake of these compounds in forensic toxicology cases were suggested.

## Supporting information


**Data S1:** Details on UHPLC‐QTOF analysis
**Data S2:** Gradient used for targeted UHPLC‐MS/MS analysis of a postmortem blood sample
**Data S3:** Extracted ion chromatogram of EPT and its in vitro metabolites A1–A7 obtained after 60 min incubation with pHLM.
**Data S4:** Extracted ion chromatogram of 4‐OH‐EPT and its in vitro metabolites B1–B7 obtained after 60 min incubation with pHLM.
**Data S5:** Extracted ion chromatogram of 5‐MeO‐EPT and its in vitro metabolites C1–C6 obtained after 60 min incubation with pHLM.
**Data S6:** Extracted ion chromatogram of 4‐OH‐EPT and its in vivo metabolites S1–S5 and B2 and B3 (zoomed in) found in a peripheral blood sample from an autopsy case.
**Data S7**: MS/MS spectra for the metabolites of EPT identified after incubation with pHLM (A1‐A7). The protonated molecules are marked by a blue tile.
**Data S8**: MS/MS spectra for the metabolites of 4‐OH‐EPT identified after incubation with pHLM (B1‐B7). The protonated molecules are marked by a blue tile.
**Data S9**: MS/MS spectra for the metabolites of 5‐MeO‐EPT identified after incubation with pHLM (C1‐C6). The protonated molecules are marked by a blue tile.
**Data S10**: MS/MS spectra for the metabolites of 4‐OH‐EPT identified in a postmortem blood sample (PM1‐PM4). The protonated molecules are marked by a blue tile.
