# Supplementary material for: Metabolite markers for three synthetic tryptamines N‐ethyl‐N‐propyltryptamine, 4‐hydroxy‐N‐ethyl‐N‐propyltryptamine, and 5‐methoxy‐N‐ethyl‐N‐propyltryptamine
Source: Drug Test Anal. 2024 Mar 9;16(12):1544–57. doi: 10.1002/dta.3668 (PMC11635065; doi:10.1002/dta.3668)
Supplement: Supplementary file 1 — Data S1: Details on UHPLC‐QTOF analysis Data S2: Gradient used for targeted UHPLC‐MS/MS analysis of a postmortem blood sample Data S3: Extracted ion chromatogram of EPT and its in vitro metabolites A1–A7 obtained after 60 min incubation with pHLM. Data S4: Extracted ion chromatogram of 4‐OH‐EPT and its in vitro metabolites B1–B7 obtained after 60 min incubation with pHLM. Data S5: Extracted ion chromatogram of 5‐MeO‐EPT and its in vitro metabolites C1–C6 obtained after 60 min incubation with pHLM. Data S6: Extracted ion chromatogram of 4‐OH‐EPT and its in vivo metabolites S1–S5 and B2 and B3 (zoomed in) found in a peripheral blood sample from an autopsy case. Data S7: MS/MS spectra for the metabolites of EPT identified after incubation with pHLM (A1‐A7). The protonated molecules are marked by a blue tile. Data S8: MS/MS spectra for the metabolites of 4‐OH‐EPT identified after incubation with pHLM (B1‐B7). The protonated molecules are marked by a blue tile. Data S9: MS/MS spectra for the metabolites of 5‐MeO‐EPT identified after incubation with pHLM (C1‐C6). The protonated molecules are marked by a blue tile. Data S10: MS/MS spectra for the metabolites of 4‐OH‐EPT identified in a postmortem blood sample (PM1‐PM4). The protonated molecules are marked by a blue tile. [file DTA-16-1544-s001.docx]

# Metabolite markers for the three synthetic tryptamines *N*-ethyl-*N*-propyltryptamine, 4-hydroxy-*N*-ethyl-*N*-propyltryptamine, and 5-methoxy-*N*-ethyl-*N*-propyltryptamine

# Short title: Metabolite markers for three synthetic tryptamines

#### Marianne Skov-Skov Bergh^1^, Inger Lise Bogen^1,2^, Katharina Elisabeth Grafinger^3^, Marilyn A. Huestis^4^, Åse Marit Leere Øiestad^5^

^1^Section for Drug Abuse Research, Department of Forensic Sciences, Division of Laboratory Medicine, Oslo University Hospital, Oslo, Norway

^2^Section for Pharmacology and Pharmaceutical Biosciences, Department of Pharmacy, The Faculty of Mathematics and Natural Sciences, University of Oslo, Oslo, Norway

^3^Institute of Chemistry and Bioanalytics, University of Applied Sciences and Arts Northwestern Switzerland, Switzerland

^4^Institute of Emerging Health Professions, Thomas Jefferson University, Philadelphia, USA

^5^Section for Forensic Toxicological Analytics, Department of Forensic Sciences, Division of Laboratory Medicine, Oslo University Hospital, Oslo, Norway

Corresponding author: Inger Lise Bogen, Address: Lovisenberggata 6, 0456 Oslo Norway, Email: rminlb@ous-hf.no

## Suppl.1: Details on UHPLC-QTOF analysis

pHLM samples and the postmortem blood sample were analyzed using a 1290 Infinity UHPLC system (Agilent Technologies, Santa Clara, CA, US) coupled to a 6550 iFunnel QTOF-MS (Agilent Technologies) operating in positive ionization mode. Chromatographic separation was achieved by an Acquity HSS T3 column (2.1 x 100 mm, 1.8 µm particles; Waters, Wexford, Ireland) fixed to an Acquity HSS T3 VanGuard pre-column (Waters) held at 65 °C. The mobile phase was composed of 10 mM ammonium formate buffer pH 3.1 (solvent A) and MeOH (solvent B) run at a 0.5 mL/min flow rate. Gradient elution with the following profile was used: 0 min; 2.5 % B, 0-14 min; 2.5-75 % B, 14-17 min; 75-100 % B, 17-19 min; 100 % B, 19-19.01 min; 100-2.5 % B, 19.01-21 min; 2.5 % B. The injection technique was flow through needle with a flexible loop and the injection volume was 4 µL.

QTOF-MS analysis was performed in auto-MS/MS mode (i.e. data dependent acquisition). The mass range was 50–1000 *m/z* (MS) with a static exclusion range of 50-122 and 650-1000 *m/z, an* acquisition rate of 6 spectra/s, and a collision energy which was 4 eV at 0 *m/z* and increased by 6 eV per 100 *m/z* when searching for parent compounds and unknown metabolites. Gas temperature was 150 °C, drying gas flow was 15 L/min, nebulizer pressure was 40 psig, sheath gas temperature and flow were 350 °C and 12 mL/min, respectively, and fragmentor voltage was 365 V. Nitrogen was employed as both desolvation and collision gas.

## Suppl.2: Gradient used for targeted UHPLC-MS/MS analysis of a postmortem blood sample

The 9.0 min gradient had the following profile: 0–0.5 min; 10 % B, 0.5–1.5 min; 10–30 % B, 1.5–2.5 min; 30 % B, 2.5-2.6 min; 30-60 % B, 2.6–5.0 min; 60–70 % B; 5.0–5.5 min; 70–90 % B, 5.5–6.7 min; 90 % B, 6.7–6.8 min; 90–98 % B, 6.8–7.5 min; 98 % B, 7.5–7.6 min; 98–10 % B, and 7.6–9.0 min; 10 % B.

##
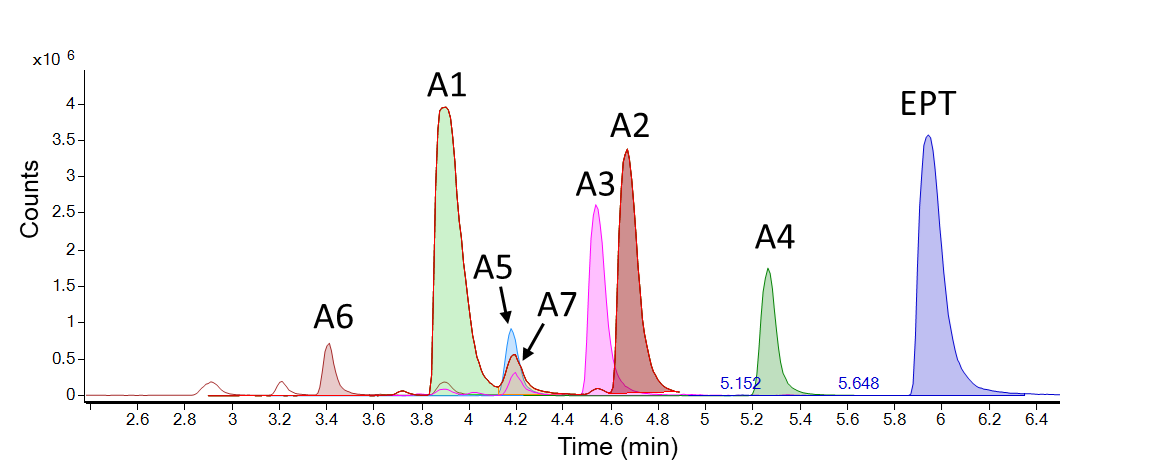
Suppl.3: Extracted ion chromatogram of EPT and its in vitro metabolites A1–A7 obtained after 60 min incubation with pHLM.

## Suppl.4: Extracted ion chromatogram of 4-OH-EPT and its in vitro metabolites B1–B7 obtained after 60 min incubation with pHLM.


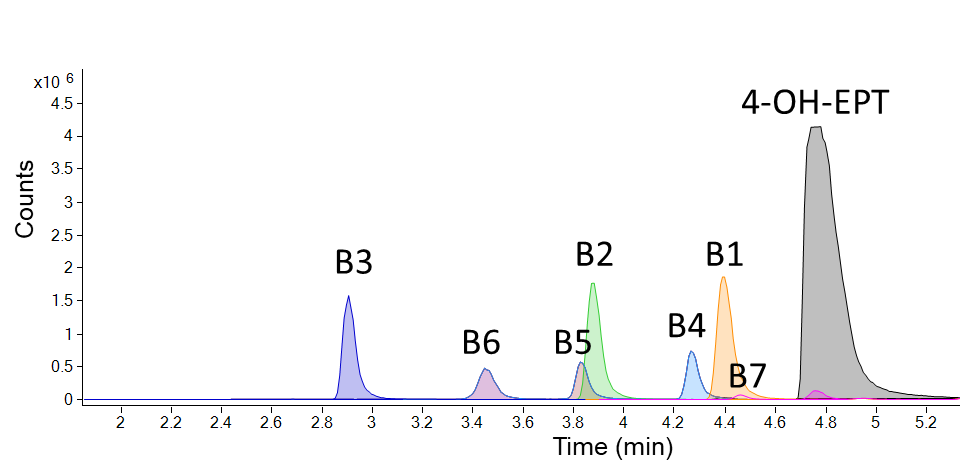


## Suppl.5: **Extracted ion chromatogram of 5-MeO-EPT and its in vitro metabolites C1–C6 obtained after 60 min incubation with pHLM.**
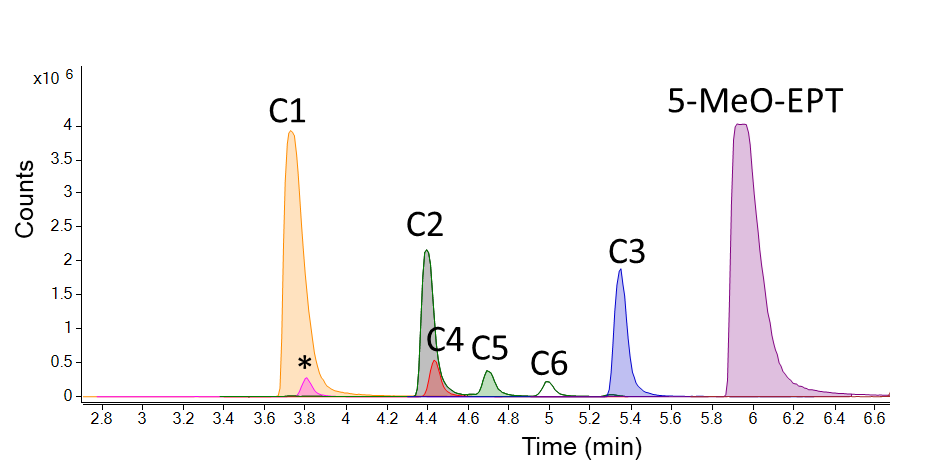
*Artefact of C1

## Suppl.6: Extracted ion chromatogram of 4-OH-EPT and its *in vivo* metabolites S1–S5 and B2 and B3 (zoomed in) found in a peripheral blood sample from an autopsy case.


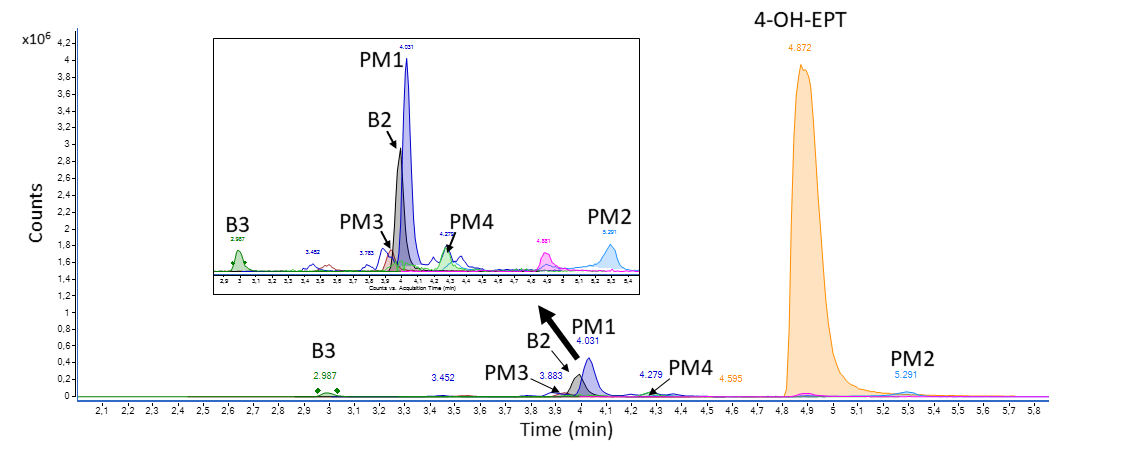


Figure S4: MS/MS for Identified Metabolites A1 and A2.

## Suppl.7**: MS/MS spectra for the metabolites of EPT identified after incubation with pHLM (A1-A7). The protonated molecules are marked by a blue tile.**

A1

202.1101

[C_12_H_14_N_2_O]^+^

dddd

_(

A3

A2

A4

A5

Figure S6: MS/MS for Identified Metabolites A5 and A6.

A6

A7

## Suppl.8: **MS/MS spectra for the metabolites of 4-OH-EPT identified after incubation with pHLM (B1-B7). The protonated molecules are marked by a blue tile.**

216.1257 [C13N2OH16]+

B1

B3

B2

Figure S9: MS/MS for Identified Metabolites B3 and B4.

B5

B4

Figure S10: MS/MS for Identified Metabolites B5 and B6.

B6

B7

## Suppl.9: **MS/MS spectra for the metabolites of 5-MeO-EPT identified after incubation with pHLM (C1-C6). The protonated molecules are marked by a blue tile.**

C1

Figure S12: MS/MS for Identified Metabolite C1 and C2.

C2

C3

C4

C4

C5

C6

## Suppl.10: **MS/MS spectra for the metabolites of 4-OH-EPT identified in a postmortem blood sample (PM1-PM4). The protonated molecules are marked by a blue tile.**

PM1

+

Structure in dotted square: exact biotransformation could not be determined.

160.0753 [C10NOH10] ^+^

PM2

176. 0704 [C10NO2H10]^+^

PM3

PM4
